# Association of house spraying with suppressed levels of drug resistance in Zimbabwe

**DOI:** 10.1186/1475-2875-3-35

**Published:** 2004-10-18

**Authors:** Sungano Mharakurwa, Susan L Mutambu, Robert Mudyiradima, Tawanda Chimbadzwa, Steven K Chandiwana, Karen P Day

**Affiliations:** 1 Department of Molecular Microbiology & Immunology, Johns Hopkins Bloomberg School of Public Health, 615 N. Wolfe Street, Baltimore MD 21205, USA; 2 The Malaria Institute at Macha, P.O. Box 630166, Choma, Zambia; 3 Blair Research Institute, P.O. Box CY 573, Causeway, Harare, Zimbabwe; 4 Provincial Medical Director (Manicaland), 24 'C' Avenue, Box 323, Mutare, Zimbabwe; 5 Postgraduate Studies School, Faculty of Health Sciences, The University of the Witwatersrand, 7 York Road, Parktown, 2193, Johannesburg, South Africa; 6 Peter Medawar Building for Pathogen Research, Department of Zoology, South Parks Road, Oxford University, Oxford OX1 3SY, UK

## Abstract

**Background:**

Public health strategies are needed to curb antimalarial drug resistance. Theoretical argument points to an association between malaria transmission and drug resistance although field evidence remains limited. Field observations, made in Zimbabwe, on the relationship between transmission and multigenic drug resistance, typified by chloroquine, are reported here.

**Methods:**

Periodic assessments of the therapeutic response of uncomplicated falciparum malaria to chloroquine in two selectively sprayed or unsprayed health centre catchments, from 1995 – 2003. Cross-sectional analysis of *in vivo *chloroquine failure events for five sites in relation to natural endemicity and spraying history.

**Results:**

During selective house spraying, the chloroquine failure rate for the sprayed catchment decreased, such that, after four years, the odds of chloroquine failure were 4× lower than before start of spraying in the area (OR 0.2, 95% CI 0.07 – 0.75, p = 0.010, n = 100). Chloroquine failure odds for the sprayed area became 4× lower than contemporaneous failure odds for the unsprayed area (OR 0.2 95% CI 0.08 – 0.65, p = 0.003, n = 156), although the likelihood of failure was not significantly different for the two catchments before selective spraying started (OR 0.5, 95% CI 0.21 – 1.32; p = 0.170, n = 88). When spraying ended, in 1999, the drug failure odds for the former sprayed area increased back 4 fold by 2003 (OR 4.2, 95%CI 1.49 – 11.78, p = 0.004, n = 146). High altitude areas with naturally lower transmission exhibited a 6× lower likelihood of drug failure than low-lying areas (OR 0.16 95% CI 0.068 – 0.353, -2 log likelihood change 23.239, p < 0.001, n = 465). Compared to sites under ongoing annual spraying, areas that were last sprayed 3–7 years ago experienced a 4-fold higher probability of chloroquine failure (OR 4.1, 95%CI 1.84 – 9.14, -2 log likelihood change 13.956, p < 0.001).

**Conclusion:**

Reduced transmission is associated with suppressed levels of resistance to chloroquine and presumably other regimens with multigenic drug resistance. It seems the adoption of transmission control alongside combination chemotherapy is a potent strategy for the future containment of drug-resistant malaria.

## Background

The escalation of parasite drug resistance has persisted as a major obstacle to malaria control for decades [1-3]. Owing to dwindling options for affordable, safe and effective drugs, rising clinical failure rates exact a substantial public health toll, especially in Africa [4,5]. In countries that recently replaced chloroquine with sulfadoxine/pyrimethamine as first line treatment, there are signs of increasing resistance to the antifolate combination [1,2,6-9]. Partly because of the spectre of drug resistance, pharmaceutical companies reduced investment in new antimalarial drug research. Fortunately, official calls in the mid 1990's led to renewed public-private sector initiatives for the development of new compounds, as well as the improvement of existing ones [10,11].

However, *Plasmodium falciparum *has repeatedly demonstrated the ability to develop resistance to practically any drug upon wider introduction, as illustrated by multi-drug resistance, especially in South East Asia [12-15]. Thus, public health strategies that delay or minimize the escalation of drug resistance are urgently required. To date, the only approach that has been widely evaluated and is currently being introduced is the use of combination chemotherapy [11,16,17] which protects constituent drugs from resistance through a multigenic mechanism of resistance and strategic pharmacological properties such as short half-life. In poor countries the effectiveness of this method is hampered by increased cost of medication. Furthermore, even the new combinations are not totally protected from the development of resistance, as illustrated by the recent confirmations of clinical failure and *in vitro *resistance to proguanil/atovaquone [18-22]. Additional strategies are, therefore, needed to ensure the successful containment of drug-resistant malaria.

Mathematical models have been proposed suggesting a relationship between malaria transmission and the evolution of drug resistance, though some workers suggest a positive association [23,24] while others propose a negative one [25,26]. Major implications for control pertain to this question. It may mean that vector control programmes are counterproductive by aggravating drug resistance, or, it could be that they complement chemotherapy by alleviating resistance. Although this interaction between transmission and drug resistance is further addressed in a review [27], the exact answer still remains uncertain against a background of limited field evidence. The present paper presents observations on the field relationship between transmission variations (both natural and vector control induced) and the levels of *in vivo *multigenic drug resistance, typified by chloroquine.

## Methods

### Study areas and population

Zimbabwe, on the southern fringes of malaria in Africa, experiences seasonal and potentially epidemic transmission characterized by a non-immune population with high probability of drug treatment [28-30]. The country has sustained a national malaria vector control programme for decades, based on intradomicilliary application of residual insecticide. From the early 1990s selective vector control was introduced, in which areas with moderate transmission are of less priority and spraying is focused in zones of high transmission/high malarial incidence. Chloroquine has remained the first line treatment for uncomplicated malaria, although a combination of chloroquine and sulfadoxine/pyrimethmanine is currently being introduced in some areas. A tiered drug distribution policy has been implemented in the country, so that, until 1997, chloroquine was the only antimalarial available at the peripheral level. Thereafter, policy revisions allowed wider distribution of sulfadoxine/pyrimethamine to treat chloroquine failure cases.

**Figure 1:**
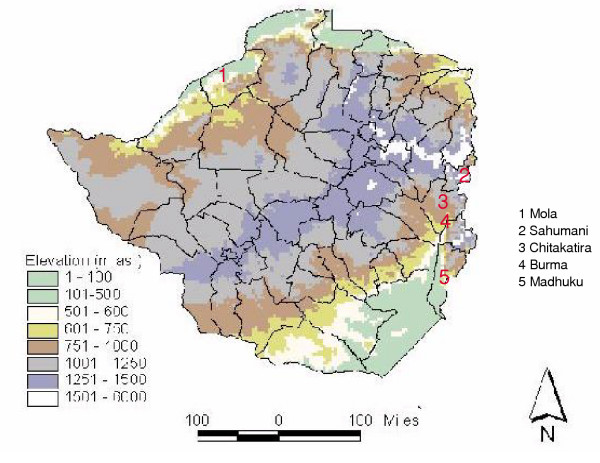
Location of study sites, shown in relation to altitudinal zones that govern malaria endemicity. Central watershed (elevation > 1200 m above sea level) experiences nil – hypoendemic malaria transmission, and endemicity increases with falling altitude towards the north and south of the country.

The study was based at five health centres located in the low-lying (<600 m above sea level) hyperendemic transmission zone as well as those in the higher altitude (600 – 1200 m asl) mesoendemic transmission zone bordering the malaria-free central watershed (Table 1, Fig 1). All the study locations experience seasonal, single peak (February-May) malaria transmission typical for Zimbabwe. Acute symptoms and complications occur across all ages in this non-immune population, where asymptomatic carriage of asexual parasitaemia is rare [28,29]. The study was conducted on uncomplicated falciparum malaria cases of all age groups presenting at the health centres for treatment.

**Table 1:** Study area characteristics

Site	Elevation	Endemicity	*Population Estimate	Villages of patient origin	Spraying status	Treatment drug
Burma Valley	683 m	mesoendemic	11764	25	Last sprayed 1999	CQ+S/P since 2001
Chitakatira	1211 m	mesoendemic	13245	28	Last sprayed 1998	CQ+S/P since 2003
Sahumani	784 m	mesoendemic	5950	24	Last sprayed 1992	CQ+S/P since 2003
Madhuku	471 m	hyperendemic	11583	39	Ongoing	CQ+S/P since 2001
Mola	≈500 m	hyperendemic	13000	28	Ongoing	CQ+SP since 2001

*2002–2003 population census.

### Study design

The study was a prospective assessment of the therapeutic response of *P. falciparum *malaria to chloroquine from 1995–2003. Consecutive assessments of therapeutic response were conducted in two mesoendemic sites during the presence or absence of selective indoor residual insecticide spraying (house spraying). Transverse assays for *Pfmdr1 *and *Pfcrt *mutations associated with chloroquine resistance were carried out in these two sites during the 1998–99 transmission season. Further assessments of *in vivo *chloroquine therapeutic response were carried out cross-sectionally in another three sites where treatment change to chloroquine (CQ) + sulfadoxine/pyrimethamine (SP) was not yet being implemented due to temporary unavailability of SP. Malarial incidence was determined retrospectively for all sites using available health centre records.

### *In vivo *antimalarial therapeutic efficacy assessments

The *in vivo *therapeutic efficacy of chloroquine was assessed using the standard WHO (1996) protocol [31]. Since this protocol was primarily targeted for regions of intense malaria transmission, two modifications were adopted to suit the seasonal/epidemic conditions of Zimbabwe. These were (i) inclusion of febrile patients of all age groups and (ii) adoption of radical asexual parasite elimination as a criterion for adequate response to treatment.

Inclusion of all age groups was on the rationale that there is no premunition in the population. Recruited patients were thus a representative sample of the symptomatic population which presents for treatment with chloroquine in the primary health care system. The radical asexual parasite elimination criterion was adopted because persistent asexual parasitaemia poses a risk of complications in non-immunes.

### Molecular detection of *Pfmdr1 *and *Pfcrt *polymorphisms

Amino acid polymorphisms at codons 86 and 1246 of the *P. falciparum Pfmdr*1 gene and at codon 76 of the *P. falciparum *chloroquine resistance transporter gene (*Pfcrt*), which are associated with chloroquine resistance [32,33], were detected by PCR and codon-specific restriction enzyme digestion [34,35]. Appropriate positive and negative control strains were used in interpretation and, except for the *Pfcrt *codon, additional restriction sites were included in the target PCR product to serve as internal controls for complete digestion.

### Ethics

The study was approved by respective provincial medical health authorities and by the Medical Research Council of Zimbabwe. Patient participation was by the informed consent of the patients themselves or guardians, in the case of children.

## Results

### Association of house-spraying with reduced levels of chloroquine resistance

#### (i) Burma Valley and Sahumani follow-up study

On the grounds of low malarial incidence, the catchments of Sahumani clinic, in Mutasa district and Burma Valley clinic, in Mutare district (Fig 1), were removed from the spraying programme in 1992, with the advent of selective control to save on inseciticide. However, the Burma catchment, which is situated on commercial farms, was re-allocated to annual spraying from 1995 – 1999 when, for economic reasons, local farmers agreed to supply the malaria control authorities with insecticide. The Burma catchment subsequently reverted to no spraying after the 1999 spraying operation, due to disagreements between commercial farmers and the government. In contrast, the Sahumani catchment, which is located in villages, remained unsprayed from 1992.

**Figure 2:**
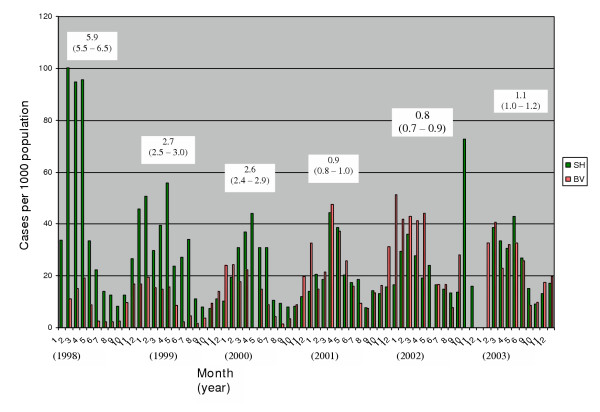
Monthly malarial incidence in Burma valley (BV) and Sahumani (SH) catchments during and after selective spraying (boxed terms, Risk Ratios (95% CI) for peak malaria transmission period (February – May)).

There were no malaria statistics for the two health centres prior to 1998, (1997 for Sahumani). However, during the selective annual spraying, the risk of contracting malaria in the sprayed Burma Valley catchment was at least 2.6 fold lower than for Sahumani (Fig 2) from 1998 – 2000. After the selective spraying operation ended in 1999, the malarial incidence became uniform for the two catchments by 2001 (Fig 2).

The therapeutic failure rate of Burma Valley decreased during selective spraying (Table 2) such that by 1999, the odds of chloroquine failure were 4× lower than they were before spraying resumed in this area (OR (95% CI) 0.233 (0.072 – 0.747), for 1999 compared to 1995; 0.482 (0.270 – 0.861), for each year of spraying: -2 log likelihood ratio change 6.432, df = 1, p = 0.011). Therapeutic failure rates were not significantly different in the Burma Valley and Sahumani catchments (1995 season) prior to selective spraying of Burma Valley (Table 2). However, by 1999 the odds of drug failure had become 4× lower in the annually sprayed Burma catchment (Table 2). The failure rate in Sahumani did not significantly change during the 4-year period (OR (95%CI): 0.62 (0.25 – 1.57), for 1998 compared to 1995; 0.53 (0.26 – 1.08) for 1999 compared to 1995; 0.85 (0.71 – 1.02) for each successive year, -2 × log likelihood ratio change, 3.080, df, 1, p = 0.0793).

**Table 2:** Chloroquine therapeutic failure (TF) rates in Sahumani and Burma Valley from 1995–2003.

	Therapeutic failure rate (n)			
	1995	1997/98*	1999	2003

Burma Valley	27.0% (37)	15.2% (33)	7.9% (93)	26.5% (83)
Sahumani	41.2% (51)	30.3% (33)	26.9% (65)	-
Odds ratio (95% CI)	1.89 (0.76 – 4.72)	2.4 (0.73 – 8.14)	4.3 (1.54 – 11.85)	-
P	0.170	0.142	0.003	-

*Burma Valley 1997 compared to Sahumani 1998.

After selective spraying ceased in 1999, the odds of drug failure in Burma valley increased back 4-fold by 2003 (OR (95%): 4.18 (1.485 – 11.782), p = 0.004, n = 146) Chloroquine efficacy assessments for 2003 were not conducted in Sahumani as the treatment was changed that year to chloroquine plus sulfadoxine/pyrimethamine.

#### (ii) *In vivo *prevalence of mutations in *Pfmdr1 *and *Pfcrt *genes

**Figure 3:**
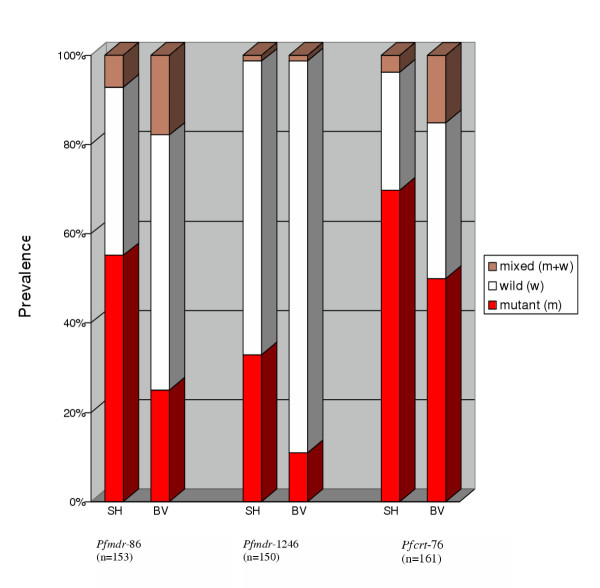
Distribution of mutated (m) and wild (w) *P. falciparum *variants in Burma Valley (BV) and Sahumani (SH), at *Pfmdr1 *codons 86 (Pfmdr-86) and 1246 (Pfmdr-1246), and *Pfcrt *codon 76 (Pfcrt-76).

Amino acid polymorphisms on *Pfmdr*1 and *Pfcrt *codons associated with chloroquine resistance were examined in pre-treatment patient samples from 1998 and 1999 in Burma Valley and Sahumani (i.e. 3–4 years after re-start of spraying in Burma Valley). Resistance-associated mutations at amino acid codons 86 and 1246 of *Pfmdr*1, and codon 76 of *Pfcrt*, were more prevalent in the Sahumani area (Table 3, Fig 3). Interestingly, mixed infections containing both mutant and wild type variants tended to be more frequent in the Burma Valley area (Fig 3), despite lower transmission in this catchment. The same distribution pattern observed with individual codons was mirrored in mutated haplotypes comprising two or more amino acid codons (Table 3). Three-codon haplotypes from the sprayed area exhibited significantly more mixed mutant and wild variants at one or more codons than corresponding haplotypes from the unsprayed area (odds ratio 5.4, 95 % CI: 1.89 – 15.54, p = 0.001, n = 131).

**Table 3:** Relative abundance of mutated *P. falciparum *genotypes in Sahumani and Burma Valley (1998 and 1999 transmission seasons).

Mutant genotype	OR (95%CI) of mutants (Sahumani : Burma Valley)	P	N
*Pfmdr1 *Tyr-86	2.4 (1.2 – 4.7)	0.013	137
*Pfmdr1 *Tyr-1246	4.2 (1.7 – 10.7)	0.001	135
*Pfcrt *Thr-76	2.2 (1.1 – 4.6)	0.028	144
*Pfmdr1 *Tyr-86 + *Pfmdr1 *Tyr-1246	3.9 (1.5 – 10.1)	0.003	132
*Pfmdr1 *Tyr-86 + *Pfmdr1 *Tyr-1246 + *Pfcrt *Thr-76	4.0 (1.6 – 10.3)	0.002	131

### Drug failure as a function of transmission

**Figure 4:**
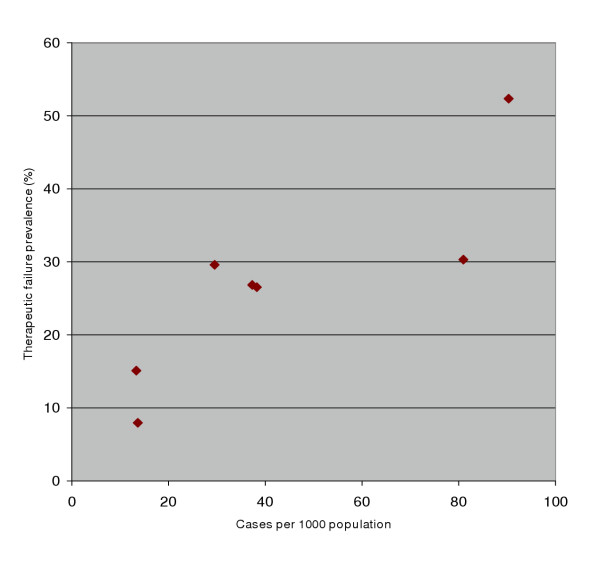
Scatter plot of therapeutic failure prevalence with malarial incidence.

**Figure 5:**
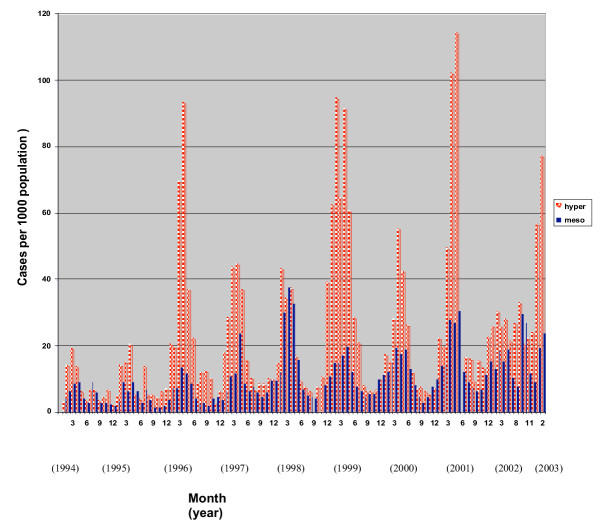
Monthly malarial incidence per thousand population in hyperendemic (hyper) and mesoendemic (meso) catchments.

A scatterplot of chloroquine therapeutic failure rate with malarial incidence suggested a positive association (Fig 4). In stead of using parametric tests on arcsine transformed data (perhaps better done with more data points), the probability of chloroquine failure was examined as a function of transmission zone, and spraying history, using a logistic model. The health centre catchments naturally falling in the mesoendemic zone according to altitudinal classifications [28], did exhibit significantly lower malarial incidence, for at least the previous 10 years, than those in the hyperendemic zone (Fig 5, Table 4). In the logistic regression, the probability of therapeutic failure was 6.4-fold lower in these mesoendemic catchments than in hyperendemic ones (Table 5, 6, 7). At any time point, catchments that were under ongoing annual spraying experienced 4-fold lower likelihood of drug failure than those that were last sprayed 3–7 years ago (Table 5).

**Table 4:** Risk of clinically diagnosed malaria in hyperendemic and mesoendemic catchments during the peak malaria season (February – May).

Year	1994	1995	1996	1997	1998	1999	2000	2001	2002	2003
Hyperendemic population (elev. <600 m)	21306	21925	22643	34298	35733	27647	25578	22651	27174	27989
Mesoendemic population (elev. ≥600 m)	65664	66497	69788	75761	88181	90910	93721	48244	96057	61625
Malarial RR (95% CI)	1.9 (1.72 – 1.99)	1.7 (1.57 – 1.81)	5.3 (5.09 – 5.55)	2.8 (2.67 – 2.88)	1.1 (1.09 – 1.17)	5.0 (4.82 – 5.12)	2.0 (1.89 – 2.04)	2.7 (2.56 – 2.75)	1.6 (1.55 – 1.69)	3.3 (3.06 – 3.48)
P	<0.001	<0.001	<0.001	<0.001	<0.001	<0.001	<0.001	<0.001	<0.001	<0.001

**Table 5:** The probability of chloroquine therapeutic failure as a function of transmission level and spraying history

: Independent variable coding					
		**Parameter coding**			

		**(1)**	**(2)**	**(3)**	**(4)**

**Study year**	**2003**	1.000	.000	.000	.000
	**1999**	.000	1.000	.000	.000
	**1998**	.000	.000	1.000	.000
	**1997**	.000	.000	.000	1.000
	**1995**	.000	.000	.000	.000
**Last annual spraying**	**3+ yrs ago**	1.000			
	**0 yrs/ongoing**	.000			
**Transmission level**	**mesoendemic**	1.000			
	**hyperendemic**	.000			

**Table 6:** The probability of chloroquine therapeutic failiure as a function of transmission level and spraying history: variables in the equation

		**B**	**S.E.**	**Wald**	**df**	**Sig.**	*Exp(B)	**95.0% C.I. for EXP(B)**	
								**Lower**	**Upper**
**Step 1(a)**	**Transmission level (1)**	-1.863	0.419	19.780	1	0.000	0.155	0.068	0.353
	**Last annual spraying (1)**	1.411	0.409	11.879	1	0.001	4.099	1.838	9.142
	**Study year**			13.037	4	0.011			
	**Study year (1)**	-0.728	0.290	6.317	1	0.012	0.483	0.274	0.852
	**Study year (2)**	-0.400	0.317	1.589	1	0.207	0.671	0.360	1.248
	**Study year (3)**	-0.224	0.440	0.259	1	0.611	0.799	0.338	1.892
	**Study year (4)**	0.262	0.445	0.346	1	0.556	1.299	0.543	3.109
	**Constant**	-0.157	0.407	0.149	1	0.700	0.855		

a Variable(s) entered on step 1: Transmission, Last annual spraying, Study year.

* corresponds to odds ratio for therapeutic failure

**Table 7:** The probability of chloroquine therapeutic failiure as a function of transmission level and spraying history: Model if term removed

**Variable**		**Model Log Likelihood**	**Change in -2 Log Likelihood**	**df**	**Sig. of the Change**
**Step 1**	**Transmission level**	-331.110	23.239	1	.000
	**Last annual spraying**	-326.469	13.956	1	.000
	**Study year**	-326.142	13.303	4	.010

## Discussion

The build up of drug-resistant *P. falciparum *malaria calls for public health strategies to maximize the useful life of antimalarials. According to the findings of the present study, reduced transmission, due to vector control or high altitude, was associated with suppressed levels of *in vivo *therapeutic failure and genotypic resistance to chloroquine. Assuming that chloroquine resistance has a multigenic mechanism, as is the general consensus [12,36,37], this association between transmission and drug resistance presumably governs other drugs or drug combinations that have polygenically encoded resistance.

From the Burma Valley and Sahumani cross-sectional assays, there was, in the sprayed catchment, a higher likelihood of infections carrying mixed mutated and wild type codons, for both *Pfcrt *and *Pfmdr1*, despite the lower transmission. This paradoxical result suggests that the sprayed area probably favoured more genetic out-crossing, resulting in recombination break down of drug-resistant haplotypes. The genetic out-crossing may partly explain the association of low drug resistance with the house spraying. Further studies are needed to verify this relationship in more areas.

In Burma Valley, despite drug pressure, the proportion of resistant parasites decreased during spraying, and subsequently resurged after the spraying stopped. This is reminiscent of the decrease in proportion of chloroquine-resistant parasites reported in China [38], and more recently in Malawi [39,40], following suspension of chloroquine use. From these observations it would seem that chloroquine-resistant parasites bear a fitness cost as drug selection advantage is removed or counteracted.

What is distinct about the current results is that the fitness cost for resistance appeared to occur in the sporogonic phase, as distinguished from an *in vivo *fitness burden that is thought to ensue following cessation of drug use. In the present results, drug selection advantage for the resistant parasites appeared to be directly counteracted by independent survival limiting factors, such as vector control and high altitude. This has important implications for control as it means that drug-resistant *P. falciparum *can be contained during drug use. Furthermore, costly acquisition of immunity in the resident population is, presumably, not the only prerequisite for curbing drug resistance.

The present results afford field evidence supporting the continuation of sustainable malaria vector control programmes. Similar findings were reported for Uganda [41], although the same workers found a difference between chloroquine (multigenic resistance) and sulfadoxine/pyrimethamine (monogenic resistance) below a critical threshold of transmission [37]. These papers may further support the findings of the present study. It has been cautioned that resistance might exacerbate as eradication is approached [42]. However, in the current study, the low transmission levels associated with high altitude and spraying showed no signs of this counterproductive effect. Moreover, in poor countries, which are the *de facto *stronghold for malaria, eradication so far remains only an academic prospect, as the malaria burden continues to increase [43]. It seems that the adoption of sustainable transmission control with combination chemotherapy is a potent approach for the future containment of drug-resistant malaria.

## Conclusions

Reduced transmission due to house spraying or high altitude is associated with suppressed levels of phenotypic and genotypic resistance to chloroquine and presumably other multigenically encoded drug regimens. Transmission control implemented with combination chemotherapy seems a potent approach for the future containment of drug-resistant malaria.

## Authors' contributions

SM was the principal investigator responsible for the study design, data collection, analysis and drafting of the manuscript. SLM carried out essential co-ordination of project activities. RM and TC afforded technical input on the manuscript and facilitated field data collection. SKC made vital contributions in original proposal development, seeking of funding, and edited the manuscript. KPD provided crucial inputs in the study design, genetic analysis, general direction and co-ordination of the study and the writing up of the manuscript.
